# Honey bee colonies act as reservoirs for two *Spiroplasma* facultative symbionts and incur complex, multiyear infection dynamics

**DOI:** 10.1002/mbo3.172

**Published:** 2014-04-28

**Authors:** Ryan S Schwarz, Érica Weinstein Teixeira, James P Tauber, Juliane M Birke, Marta Fonseca Martins, Isabela Fonseca, Jay D Evans

**Affiliations:** 1 Bee Research Lab, U.S. Department of AgricultureBARC-East Bldg. 306, 10300 Baltimore Ave., Beltsville, Maryland, 20705; 2 Honey Bee Health Laboratory (LASA), São Paulo State Agribusiness Technology Agency (APTA)SAA-SP, PO Box 07, 12.400-970 Pindamonhangaba, São Paulo, Brazil; 3 Molecular Genetics Laboratory, Embrapa Dairy Cattle, Rua Eugênio do Nascimento610, CEP 36.038-330 Juiz de Fora, MG, Brazil

**Keywords:** Flower microbe communities, host–parasite interaction, microbial prevalence, spiroplasma, symbiont, temporal survey

## Abstract

Two species of *Spiroplasma* (Mollicutes) bacteria were isolated from and described as pathogens of the European honey bee, *Apis mellifera*, ∼30 years ago but recent information on them is lacking despite global concern to understand bee population declines. Here we provide a comprehensive survey for the prevalence of these two *Spiroplasma* species in current populations of honey bees using improved molecular diagnostic techniques to assay multiyear colony samples from North America (U.S.A.) and South America (Brazil). Significant annual and seasonal fluctuations of *Spiroplasma apis* and *Spiroplasma melliferum* prevalence in colonies from the U.S.A. (*n* = 616) and Brazil (*n* = 139) occurred during surveys from 2011 through 2013. Overall, 33% of U.S.A. colonies and 54% of Brazil colonies were infected by *Spiroplasma* spp., where *S. melliferum* predominated over *S. apis* in both countries (25% vs. 14% and 44% vs. 38% frequency, respectively). Colonies were co-infected by both species more frequently than expected in both countries and at a much higher rate in Brazil (52%) compared to the U.S.A. (16.5%). U.S.A. samples showed that both species were prevalent not only during spring, as expected from prior research, but also during other seasons. These findings demonstrate that the model of honey bee spiroplasmas as springtime-restricted pathogens needs to be broadened and their role as occasional pathogens considered in current contexts.

## Introduction

Attempts to identify pathogens associated with unusually large numbers of moribund or dead worker honey bees *Apis mellifera* L. (Hymenoptera: Apidae) outside of colonies in the U.S.A. (Clark 1977) and France (Mouches et al. 1982, 1984) revealed heavy loads of two species of cultivable bacteria within the class Mollicutes (Entomoplasmatales: Spiroplasmataceae) during hemolymph and gut tissue examinations from diseased bees. These bacteria tended to flourish in adult honey bees specifically during spring then vanish by summer from available serologic and microscopic detection, suggesting they opportunistically infected honey bee colonies between spring and summer via transmission from other hosts. Formally described and denominated for their host as *Spiroplasma apis* (Mouches et al. 1983) and *Spiroplasma melliferum* (Clark et al. 1985), they were tentatively identified as the causative agents of neurological disease in bees specifically during the spring using the terms “spiroplasmosis” and “May disease.”

**Figure 1:**
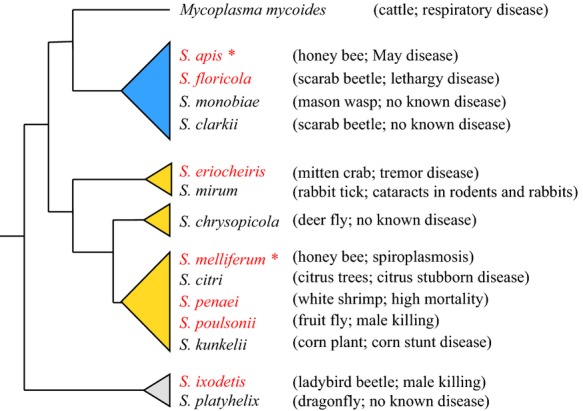
Spiroplasmas are a monophyletic group in the class Mollicutes with strong support of three major clades. Honey bee *Spiroplasma* spp. (indicated by *) arise within two distinct clades: the Apis clade (blue) and the Citri clade (gold). Species described previously as arthropod pathogens are in red font. Putative primary hosts and associated disease, if any, are given. Phylogeny based on (Gasparich 2004; Bi et al. 2008) from 16S and 23S rRNA data.

Spiroplasmas are a monophyletic group of bacteria that have unique cytoplasmic membrane proteins (i.e., spiralin) (Razin et al. 1998) and cytoskeletal architecture that enables rapid motility of their helical form through liquid substrates without the use of flagella or cilia (Trachtenberg et al. 2003; Shaevitz et al. 2005). The two honey bee *Spiroplasma* spp. represent two of three major *Spiroplasma* clades (Gasparich 2004; Bi et al. 2008) (Fig. 1), the Apis clade (*S. apis*) and Citri clade (*S. melliferum*). All three clades include etiologic agents of disease in arthropods, with additional species that are well known plant pathogens. Within arthropods, spiroplasmas may live both intracellularly and systemically via the hemolymph, with corresponding differences in pathology (Dowell et al. 1981; Clark and Whitcomb 1984; Eskafi et al. 1987) and mortality (Mouches et al. 1984; Nunan et al. 2004). While directly harmful consequences to honey bees from *S. apis* are supported (Mouches et al. 1982, 1983), the effects of *S. melliferum* infections are less clear, but purportedly lead to early mortality when orally administered to adult bees (Clark 1977, 1978; Clark and Whitcomb 1984). Additional arthropod diseases attributed to spiroplasmas include tremor disease in crabs (*Eriocheir sinensis*; Wang et al. 2004, 2011), lethargy disease in beetles (*Melolontha melolontha*) (Dowell et al. 1981; Clark et al. 1982; Eskafi et al. 1987), and sex-ratio disorders due to significantly increased mortality of developing males in populations of fruit flies (Hackett et al. 1986; Williamson et al. 1999), butterflies (Jiggins et al. 2000), and beetles (Hurst et al. 1999; Majerus et al. 1999). Although a number of spiroplasma symbionts studied in detail are detrimental to their hosts, others appear to act as mutualists (Ebbert and Nault 2001; Jaenike et al. 2010).

The first *Spiroplasma* species identified in honey bees (Clark 1977), denominated *S. melliferum* strain BC-3 (Clark et al. 1985), was isolated in Maryland, U.S.A. and anecdotally implicated in high colony mortality during the spring of 1977. Prevalence in moribund/dead bees (25–100%) collected from declining colonies was notably higher than in foragers from apparently healthy colonies (0–37%) (Clark 1978) and spiroplasmas were not detected from this location in the previous spring when colony mortalities were apparently normal (Clark 1977). Additional factors to these colony mortalities cannot be ruled out as no further descriptions were given related to colony health status and honey bee colony declines have been associated with a diversity of biotic (Cox-Foster et al. 2007; Cornman et al. 2012) and abiotic threats (van Engelsdorp et al. 2009).

Shortly following the discovery of *S. melliferum*, researchers in France studying a syndrome locally called “May disease” by apiarists due to its typical springtime occurrence (May–June), isolated a *Spiroplasma* species serologically distinct from *S. melliferum* in 1981 (Mouches et al. 1982) that was later denominated *S. apis* strain B31 (Mouches et al. 1983). This species was determined to be the etiologic agent that caused adults to become unable to fly and “quiver and creep…and often move in small groups to sites at some distance from the affected hives, where they die” (Mouches et al. 1984). Hemolymph examined from diseased worker bees showed high *S. apis* loads and “many thousands” of bee mortalities over a short time (4–5 days) from a single apiary that typically did not result in the immediate demise of the entire colony. Diminished colony productivity in the form of a >25% decrease in honey production was also attributed to *S. apis* (Mouches et al. 1982).

Synchronized occurrence of *S. melliferum* with peak plant flowering periods led to the hypothesis that flowers were reservoirs for these bacteria (Clark 1978, 1982). This hypothesis was supported by the isolation of both *S. melliferum* (Clark 1978; Davis 1978) and *S. apis* (Mouches et al. 1984) from flowers prominently used by honey bees during spring and/or summer. However, additional isolates serologically distinct from the honey bee isolates were also reported (Davis 1978; Raju et al. 1981; Mouches et al. 1984), supporting current understanding that flowers, particularly nectar, may harbor diverse microbial communities (e.g., Fridman et al. 2012; Jacquemyn et al. 2013) from which bees and other pollinators can become inoculated (McFrederick et al. 2012). Presumably, honey bees became infected from these reservoir plants and then either died or cleared the infections once the peak flowering period was over. The absence of detectable spiroplasmas from flowers outside of spring and summer and an unknown mode of maintenance during the dormant wintering period in these regions, however, left a puzzling gap in the hypothesis that plants in temperate climates were reservoirs for *S. apis* and *S. melliferum* from which honey bees opportunistically became infected every spring (Clark 1977; Whitcomb 1981). Speculatively, longer flowering times in tropical and subtropical climates may extend the spiroplasma transmission cycle to honey bees yet periods of minimal flowering, as normally occurs during drought cycles, brings into question if and how spiroplasma populations survive if plants are the reservoirs.

Seasonality can cause broad variation in the spread and persistence of a pathogen in its host (Altizer et al. 2006), creating complex multiyear dynamics between the host and pathogen. Such a case may apply to *S. apis* and *S. melliferum* and explain the inconsistent (though sparse) reports of impacts on honey bee colonies. However, since original research published ∼30 years ago, no study has addressed the life history and status of these two species in modern managed honey bees until the work presented here. Recently, two species of bumblebee (*Bombus*) in Europe (Meeus et al. 2012) were reported to carry *S. apis* (*Bombus pratorum*) and *S. melliferum* (*B. pratorum* and *B. pascuorum*) using multiplex PCR amplification diagnostics. Frequency, load, or seasonal distribution patterns in bumblebees have not been addressed and it is unknown whether this report represents rare spillover infections or if *S. apis* and *S. melliferum* regularly use multiple hosts (honey bee and bumblebee).

Clarifying the infection dynamics of both *S. apis* and *S. melliferum* in current bee populations, including temporal and geographic distribution and prevalence patterns, will provide a step toward understanding the life history dynamics of these bacteria and their potential for impacting bee host populations (Potts et al. 2010). Toward this aim, we applied modern, highly sensitive molecular diagnostics and report the frequency and seasonal profiles for both *S. apis* and *S. melliferum* using multiyear data (2011–2013) in two geographically unique habitats, the U.S.A. in North America and Brazil in South America. We describe a quantitative real-time PCR (qPCR) assay that specifically detects *S. apis* (to 10 genome copies) and *S. melliferum* (to 100 genome copies). This assay is suitable for individual analyses or whole colony surveys for honey bee microbes using pooled samples of bees (Evans et al. 2013). Our findings support that *S. apis* and *S. melliferum* are facultative symbionts and not part of the typical honey bee microbiota, but may be temporally and regionally common and thus influential to honey bee health and disease cycles.

## Materials and Methods

### Apiaries and bee collections

#### U.S.A. samples

Honey bee colonies founded by Italian queens (*Apis mellifera ligustica)* were maintained in multiple small (<20 colonies) apiaries at the Beltsville Agricultural Research Center (BARC) in Maryland, U.S.A. located within 8 km of one another. Individual colonies were randomly marked at the beginning of the study and were continually sampled at different times of year until they either died, were withdrawn from the survey due to other research needs, or until the end of the survey was reached. New colonies were added to the survey as needed (almost entirely following colony mortalities) to obtain and maintain desired sample size, usually during spring (April) when new colonies were established within apiaries via packages from Georgia, U.S.A. and local colony splits. Adult bees were collected within colonies from frames or inner covers to obtain a mixed sample of mature worker development stages (house bees and foragers). Sample tubes of bees were immediately buried in ice and held <2 h until transport to the laboratory where they were stored at −80°C.

#### Brazil samples

Forager bees were collected at the entrance of the hive and workers covering frames from Africanized (*A. m. scutellata* × European *A. m*. subspecies) honey bee colonies by the official Veterinary Services of the states in Brazil or directly by researchers from 11 states across the country (Fig. S1) spanning a longitudinal gradient of roughly 30° (∼4060 km). Samples were collected randomly according to accessibility as part of a national effort to assess honey bee microbes from throughout Brazil and preserved in 70% ethyl alcohol. Samples were processed at the Honey Bee Health Laboratory (LASA)/São Paulo State Agribusiness Technology (APTA). The majority of samples (116 of 139) were obtained within a more limited geographic range (∼700 km) from two states in Brazil: Santa Catarina (SC), with 16 municipalities sampled and São Paulo (SP) with 12 municipalities sampled.

### Nucleic acid extraction from pooled honey bee samples

#### U.S.A. samples

Standard protocol for colony surveys in the U.S.A. laboratory follows the “bulk extraction of RNA from 50 to 100 whole bees using the acid-phenol method” as detailed in Evans et al. (2013) using 50 frozen (−80°C) workers from each colony. Air-dried RNA pellets were resuspended in 200 *μ*L nuclease-free water with a 10-min incubation at 55°C. Following spectrophotometer RNA quantification, 1.5 *μ*g of total RNA, determined using a NanoDrop ND-8000 spectrophotometer (NanoDrop Technologies, Wilmington, DE), were transferred to a 0.5 mL microtube and treated with 1 U DNase I (Ambion, Austin, TX) and 5 mmol/L ethylene diamine tetraacetic acid (EDTA) for 30 min at 37°C followed by heat inactivation at 75°C for 10 min. First-strand cDNA synthesis using both random and oligo(dT)_12-18_ primers and 20 U of RNase OUT (Invitrogen; Carlsbad, CA) ribonuclease inhibitor was performed as per manufacturer's recommendation with SuperScript II reverse transcriptase (Invitrogen). Completed cDNA reactions were diluted with nuclease-free water 1:9, resulting in a final concentration of ∼8 ng/*μ*L.

#### Brazil samples

Brazil samples were processed according to standard DNA extraction protocol independently implemented in Brazil for microbial analysis of pooled honey bees. Whole honey bees (10–30 per colony) were macerated with a sterile mortar and pestle in 1 mL of sterile distilled water per bee for ∼1 min. The macerate was filtered through a sterile cotton pad and the eluate was centrifuged at 2518*g* for 40 min at room temperature. We note that this speed is below that generally recommended by the American Type Culture Collection (ATCC) for axenic *Spiroplasma* cultures (∼4500*g*). The supernatant was discarded and the pellet was resuspended in 1 mL of sterile water. This suspension was centrifuged at 10,000*g* for 5 min, after which the supernatant was discarded and the pellet was submitted for DNA extraction employing a Qiagen DNeasy® Plant Mini Kit (Qiagen, Hilden, Germany), according to the manufacturer's recommendations. Sample concentrations were determined with a NanoDrop ND-1000 spectrophotometer (NanoDrop Technologies).

### Bacterial cultures

For reference controls and primer optimization, we cultured *S. apis* B31 (ATCC 33834) and *S. melliferum* BC-3 (ATCC 33219) using medium M1D (Whitcomb 1983) aerobically at 30°C. Cells were pelleted and washed from growth media prior to gDNA isolation using the following extraction buffer: 2% (w/v) hexadecyltrimethylammonium bromide (CTAB); 100 mmol/L Tris-HCl (pH 8); 1.4 mol/L NaCl; 20 mmol/L EDTA; 0.2% 2-mercaptoethanol; 50 *μ*g proteinase K (Promega, Madison, WI); 5% v/v of RNase cocktail (Ambion). Homogenization was conducted at 60°C for 3 h followed by phenol:chloroform:isoamyl alcohol (25:24:1) DNA extraction, 100% isopropyl alcohol and 0.3 mol/L sodium acetate precipitation, and 75% ethanol wash. Purified DNA was diluted in nuclease-free water, quality verified by 1% agarose gel electrophoresis and spectrophotometer analysis, and stored at −20°C.

### Primers for qPCR analyses and recombinant clones

GenBank accession AY736030 of *S. apis* strain B31 partial 16S–23S rRNA sequence was aligned against homologous regions from other *Spiroplasma* species in the Apis and Citri clades (Fig. S2) using MUSCLE (Edgar 2004). Variable regions were targeted for qPCR primer design, resulting in the species-specific primer “*S. apis* ITS forward”: 5′-AATGCCAGAAGCACGTATCC-3′ and “*S. apis* ITS reverse”: 5′-GAACGAGATATACTCATAAGCTGTTACAC-3′. Optimal annealing temperature of 60°C produced a 190 bp amplicon from the 3′ end of 16S rRNA to the ITS-1 region (Table S1 and Fig. S3). A primer set designed for multiplex PCR (Meeus et al. 2012) to specifically target a *spiralin*-like gene of *S. melliferum* (GenBank accession M59366) was adapted and worked well in our qPCR analyses, “Ms-160 forward”: 5′-TTGCAAAAGCTGTTTTAGATGC-3′ and “Ms-160 reverse”: 5′-TGACCAGAAATGTTTGCTGAA-3′. An annealing temperature of 60°C produced a 160 bp amplicon (Table S1 and Fig. S3). *Apis mellifera* ribosomal protein S5 (RPS5) forward: 5′-AATTATTTGGTCGCTGGAATTG-3′ and reverse: 5′-TAACGTCCAGCAGAATGTGGTA-3′ qPCR primers (Evans 2006) were run for each sample to assess template quality. Species specificity for each *Spiroplasma* primer set was verified using control *S. apis* and *S. melliferum* templates. Amplicons from positive control templates for each *Spiroplasma* species target were cloned into pGEM-T Easy vectors (Promega) as per manufacturer's guidelines.

### qPCR analyses

qPCR in the U.S.A. was performed in 96-well plate format on the CFX96 real-time system (Bio-Rad, Hercules, CA) in 5 *μ*L total volume for each reaction with 1× SsoFast EvaGreen supermix (Bio-Rad), 150 nmol/L of each forward and reverse primer for a given target, and 1 *μ*L (∼8 ng) of cDNA using the following cycling conditions: 97°C for 1 min; 50 cycles of 95°C for 2 sec and 60°C for 5 sec; melt curve from 65 to 95°C at 0.5°C/5 sec increments. qPCR in Brazil used SYBR Green PCR Master Mix kit (Applied Biosystems, Foster City, CA) with 1 *μ*L DNA template, 100 nmol/L each primer, and 1× PCR Master Mix in 25 *μ*L final volume using a 7300 Real-Time PCR System (Applied Biosystems). Amplification conditions were: 2 min at 50°C, 10 min at 95°C, 40 cycles of 15 sec at 95°C and 1 min at 60°C. Both laboratories ran duplicate technical replicates as well as dissociation curves and no template controls to monitor amplification integrity. Each nucleic acid sample was analyzed using three primer pairs: *S. apis* ITS, Ms-160, and RPS5.

Standard curves were run using recombinant plasmid dilution series of the primer targets from 10^1^ to 10^8^ copies to assess primer sensitivity to target copy number available in qPCR reaction (Fig. S4A). Expected spiroplasma amplicon dissociations at 81.5°C for “*S. apis* ITS” and 78°C for “Ms-160” (Fig. S4B) as well as 75.5°C for “RPS5” (Schwarz and Evans 2013) were verified for every positive sample in all data sets. Dissociation curves differed by −2°C under conditions at the laboratory in Brazil compared to the U.S.A. laboratory as expected due to different qPCR reagents and conditions: “*S. apis* ITS” = 79.5°C, “Ms-160” = 76°C. Laboratories in both the U.S.A. and Brazil independently confirmed the expected target for each primer pair from experimental samples using gel electrophoresis (Fig. S3) and BigDye® Terminator v3.1 automatic Sanger sequencing (Table S1) of amplicons.

### Statistical analyses

Unless otherwise described, data were imported and analyzed using Prism™ 5 for Mac OS X (GraphPad Software, Inc., La Jolla, CA). Sample data were grouped for seasonal analyses based on their collection dates according to winter/summer solstice and vernal/autumnal equinox. Overall prevalence of *Spiroplasma* spp. in the U.S.A. versus Brazil was compared using Gaussian approximation with two-tailed Mann–Whitney *U* test, whereas overall variation in standard deviations or the proportions were tested using two-tailed unpaired *t* test with Welch's correction and *F* test. Contingency table analyses were performed using the number of honey bee colonies with or without specific spiroplasma infections. To test the null hypothesis that numbers of colonies (infected vs. not infected) were equal across all years and across all seasons, *P* values were obtained using chi-square tests (Table 1). To test for equal numbers of infected versus not infected colonies among different years (Table 2) and among different seasons (Table 3), *P* values were calculated using two-tailed Fisher's exact tests. Chi-square goodness-of-fit tests of seasonal sampling efforts were calculated using observed sample numbers in each season versus an expected even distribution for 1.0 total expected proportions using http://vassarstats.net/index.html (Lowry 2014). To assess the observed versus expected prevalence of co-infections, two-tailed exact binomial probabilities were calculated using http://vassarstats.net/index.html (Lowry 2014), where *n* is the number of colonies sampled, *k* is the number of colonies with both *S. apis* and *S. melliferum* (co-infection), *P* is the expected probability of co-infections occurring (calculated as the product of observed individual species frequency), and *q* is the expected probability of co-infections not occurring (calculated as 1 − *P*). Note that we present both the arithmetic mean (prevalence) and weighted mean (mean prevalence ± SEM).

**Table 1:** Contingency analyses (by year and season) of four spiroplasma infection categories from *Apis mellifera* colonies sampled during 2011 to 2013 in the U.S.A. and Brazil

	U.S.A. (Maryland)			Brazil (11 states)			Brazil (SC and SP only)		
			
Variable	*χ*^2^	df	*P*-value^2^	*χ*^2^	df	*P*-value^2^	*χ*^2^	df	*P*-value^2^
*Spiroplasma* spp.^1^									
Year	20.58	2	**<0.0001**	17.32	2	**0.0002**	12.20	2	**0.0022**
Season	17.40	3	**0.0006**	6.07	2	**0.0480**	16.39	2	**0.0003**
*S. apis*									
Year	27.34	2	**<0.0001**	9.76	2	**0.0076**	7.90	2	**0.0193**
Season	0.45	3	0.9294	8.74	2	**0.0126**	9.23	2	**0.0099**
*S. melliferum*									
Year	5.82	2	0.0544	16.70	2	**0.0002**	14.42	2	**0.0007**
Season	29.92	3	**<0.0001**	3.25	2	0.1966	10.73	2	**0.0047**^4^
Co-infections^3^									
Year	4.87	2	0.0875	2.62	2	0.2704	2.29	2	0.3186
Season	15.38	3	**0.0015**	2.22	2	0.3290	0.06	1	0.8146

Brazil data were analyzed across broad (11 states) and narrowed (SC and SP only) geographies to account for longitudinal variables. SC, Santa Catarina; SP, São Paulo.

1 Infected by *S. apis* and/or *S. melliferum*.

2 Significant tests at *α* < 0.05 in bold font, using chi-square test.

3 Colonies infected by both species versus single species.

4 Unique finding when Brazil data were delimited by geography.

**Table 2:** Contingency analyses of annual spiroplasma infections in *Apis mellifera* colonies sampled between 2011 and 2013 from the U.S.A. and Brazil

		*Spiroplasma* spp.^1^	*Spiroplasma apis*	*Spiroplasma melliferum*
Year		*P*-value^2^	*P*-value^2^	*P*-value^2^
U.S.A. (Maryland)				
2011	vs. 2012	**0.0003**	**0.0005**	**0.0449**
	vs. 2013	**0.0002**	**<0.0001**	0.0567
2012	vs. 2013	0.2325	**0.0060**	0.6693
Brazil (11 states)				
2011	vs. 2012	0.3842	0.3246	0.3246
	vs. 2013	**0.0168**	0.1619	**0.0338**
2012	vs. 2013	**0.0002**	**0.0026**	**0.0001**
Brazil (SC and SP only)				
2011	vs. 2012	0.5084	0.4633	0.6857
	vs. 2013	**0.0424**	0.2606	**0.0465**
2012	vs. 2013	**0.0008**	**0.0094**	**0.0008**

Brazil data were analyzed across broad (11 states) and narrowed (SC and SP only) geographies, although no significant differences based on geographic variables occurred. SC, Santa Catarina; SP, São Paulo.

1 Infected by *S. apis* and/or *S. melliferum*.

2 Significant tests at *α* < 0.05 in bold font, using two-tailed Fisher's exact test.

**Table 3:** Contingency analyses of seasonal spiroplasma infections in *Apis mellifera* colonies between 2011 and 2013 in the U.S.A. and Brazil

		Spiroplasma spp.^1^	*Spiroplasma apis*	Spiroplasma melliferum
Season		*P*-value^2^	*P*-value^2^	*P*-value^2^
U.S.A. (Maryland)				
Spring	vs. Winter	**0.0154**	1.0000	**0.0103**
	vs. Summer	**0.0058**	0.5427	**0.0036**
	vs. Fall	**0.0006**	0.7019	**<0.0001**
Winter	vs. Summer	0.7003	0.7385	0.7759
	vs. Fall	0.1519	0.8390	**0.0011**
Summer	vs. Fall	0.2476	1.0000	**0.0042**
Brazil (11 states)				
Fall	vs. Winter	**0.0335**	**0.0025**	0.1932
	vs. Spring	0.2834	0.7887	0.2837
Spring	vs. Winter	0.4813	**0.0391**	1.0000
Brazil (SC and SP only)				
Fall	vs. Winter	**<0.0001**	**0.0022**	**0.0008**^3^
	vs. Spring	0.5322	1.0000	0.5260
Spring	vs. Winter	**0.0062**^3^	**0.0351**	0.0902

Broad (11 states) and narrowed (SC and SP only) analyses were performed on Brazil samples to assess geography as a variable. SC, Santa Catarina; SP, São Paulo.

1 Infected by *S. apis* and/or *S. melliferum*.

2 Significant tests at *α* < 0.05 in bold font, using two-tailed Fisher's exact test.

3 Unique finding when Brazil data were delimited by geography.

## Results

### Sampling effort in the U.S.A. and Brazil

**Figure 2:**
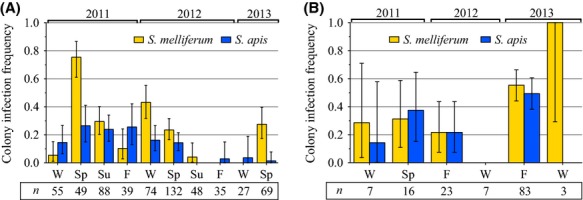
Infection frequency profiles of *Spiroplasma apis* (blue) and *S. melliferum* (gold) from honey bee (*Apis mellifera*) colonies in (A) Maryland, U.S.A. (*n* = 616) and (B) 11 states from Brazil (*n* = 139) between 2011 and 2013. Species-specific primers were used in qPCR to determine infection frequency from pooled samples of adult honey bees (*n*) collected by season: winter (W), spring (Sp), summer (Su), fall (F). Absence of a column indicates no detection of that species. Error bars show 95% binomial confidence intervals.

Between 2011 and 2013, 616 honey bee colony samples were collected and processed (see “Materials and Methods”) in Maryland, U.S.A. at small, nonmigratory apiaries located no more than 8 km from one another. The number of colonies sampled in each year varied (*χ*^2^ = 95.52, df = 2, *P* < 0.0001): 231 (37.5%) in 2011, 289 (46.9%) in 2012, and 96 (15.6%) in 2013. Prior to data analyses, samples were grouped according to season in which they were collected (Fig. 2A). Colonies were sampled during all four seasons in 2011 and 2012, but only during winter and spring in 2013. Seasonal sampling efforts were: 25.3% (*n* = 156) from three winter periods, 40.6% (*n* = 250) from three spring periods, 22.1% (*n* = 136) from two summer periods, 12.0% (*n* = 74) from two fall periods. Seasonal sampling effort was not evenly distributed (*χ*^2^ = 103.5, df = 3, *P* < 0.0001), with significantly more samples collected in spring (+62.3% from expected) and significantly fewer in fall (−52.0% from expected).

During these same 3 years, an independently initiated survey of honey bee colonies in Brazil collected and processed 139 colony samples (see “Materials and Methods”) from 11 different states (Fig. S1), although most samples were taken in the states of São Paulo (SP, *n* = 77) and Santa Catarina (SC, *n* = 39). All remaining states (Bahia, Ceará, Goiás, Minas Gerais, Mato Grosso do Sul, Paraná, Rio de Janeiro, Rio Grande do Norte, and Rio Grande do Sul) were each represented by ≤5 colony samples (Table S2). The number of samples collected varied significantly by year, with 23 (16.5%) in 2011, 30 (21.6%) in 2012, and 86 (61.9%) in 2013 (*χ*^2^ = 51.47, df = 2, *P* < 0.0001). Samples were collected from two seasons each year (Fig. 2B) to include all seasons during the study except summer. In total, samples were grouped according to season based on their collection date with the following distribution: 12.2% from three winter periods (*n* = 17), 11.5% from two spring periods (*n* = 16), and 76.3% from two fall periods (*n* = 106). Seasonal sampling effort was biased for fall (+129% from expected) and against winter and spring (−63% and −65% from expected, respectively) (*χ*^2^ = 115.27, df = 2, *P* < 0.0001).

### Total *Spiroplasma* annual and seasonal prevalence

**Figure 3:**
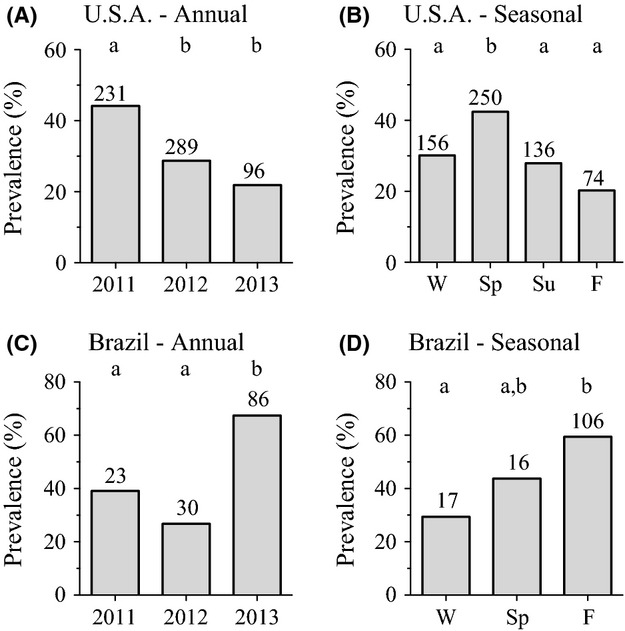
Summarized data for overall *Spiroplasma* spp. (*S. apis* and/or *S. melliferum*) prevalence in honey bee colonies sampled from 2011 through 2013 by year and by season show significant annual and seasonal variation occurred in both Maryland, U.S.A (A) and 11 states in Brazil (C). Distinct seasonal patterns in prevalence of *Spiroplasma* spp. populations occurred in the U.S.A. (B) compared to Brazil (D). Significant differences in prevalence at *α* < 0.05 (indicated by columns with different letters) were determined with two-tailed Fisher's exact tests. Spiroplasma were detected by qPCR from pooled samples of adult worker honey bees (*Apis mellifera*).

Year to year and seasonal variation in prevalence (proportion of colonies infected) of all *Spiroplasma* spp. (*S. apis* and/or *S. melliferum*) from U.S.A. colonies was evident (Fig. 2A) and the null hypotheses that prevalence was the same from year to year (*P* < 0.0001) and season to season (*P* = 0.0006) were rejected by contingency analyses (Table 1). Overall, U.S.A. honey bee colonies had a 33.4% prevalence rate for *Spiroplasma* spp. between 2011 and 2013 (206 of 616 colony samples; mean 31.6 ± 6.6% SEM; Table S3). Prevalence was significantly higher during 2011 (44.2%) compared to 2012 (28.7%) (*P* = 0.0003; Fig. 3A and Table 2). A significant difference in prevalence also occurred between 2011 and 2013 (*P* = 0.0002), however, only data from the first half of the year were collected in 2013, which limits direct comparison to other years.

Contingency analyses on seasonal data from U.S.A. colonies (Table 3 and Fig. 3B) showed *Spiroplasma* spp. prevalence was significantly higher only during spring (42.4%; mean 48.9 ± 17.4% SEM) compared to other seasons (*P* = 0.0154, *P* = 0.0058, *P* = 0.0006). We note that the lowest proportion of colonies, 20.3%, were infected during fall, with winter and summer fairly similar to each other at 30.1% and 27.9%, respectively. Tabulated versions of all data for annual and seasonal *Spiroplasma* spp. prevalence analyses in honey bee colonies from the U.S.A. and Brazil are available in Table S3.

Populations of *Spiroplasma* spp. in honey bee colonies from Brazil were similarly dynamic (Fig. 2B) with unequal year to year (*P* = 0.0002) and season to season (*P* = 0.0480) prevalence (Table 1). Overall prevalence of *Spiroplasma* spp. in Brazil between 2011 and 2013 was 54.0% (75 of 139 colonies; mean 44.4 ± 12.1% SEM; Table S3). Despite the 20.6% overall higher *Spiroplasma* spp. prevalence in Brazil compared to the U.S.A., mean prevalence and variation (standard deviation) did not differ significantly between these sites (Mann–Whitney *U* test: *P* = 0.4923, *U* = 23.00; *F* test of variance: *P* = 0.3646, *F* = 1.907, df = 5.9). Contingency analyses (Table 2 and Fig. 3C) showed *Spiroplasma* spp. prevalence in Brazil during 2013 (67.4%) was significantly higher than 2011 (39.1%; *P* = 0.0168) and 2012 (26.7%; *P* = 0.0002). To account for potential variation due to geographic influences in colonies across the 11 states in Brazil, we also analyzed a regionally delimited subset of samples (*n* = 116) from two states (SC and SP), which corroborated the significant annual and seasonal variation in overall *Spiroplasma* spp. prevalence (Table 1, Brazil – SC and SP only) and corroborated the significantly higher comparative prevalence in 2013 (Table 2, Brazil – SC and SP only; *P* = 0.0424 and *P* = 0.0008).

The seasonal distribution of *Spiroplasma* spp. in colonies from Brazil showed unique overall prevalence patterns compared to the U.S.A. during 2011 to 2013, with highest prevalence detected during fall (59.4%) instead of spring as in the U.S.A. (Fig. 3D). Total *Spiroplasma* spp. prevalence in Brazil during fall was significantly higher (*P* = 0.0335; mean = 50.6 ± 15.8% SEM) than winter (29.4%; mean = 42.9 ± 29.7% SEM) but did not differ significantly from spring (43.8%; Table 3). When colonies from SC and SP only were analyzed, where seasonal temperature variation is greater than more equatorial states in Brazil, *Spiroplasma* spp. prevalence was significantly higher in fall (*P* < 0.0001) and spring (*P* = 0.0062) compared to winter (Table 3). This was driven by higher springtime prevalence (54.6% vs. 43.8%) and lower winter prevalence (0% vs. 29.4%) in SC and SP compared to all of Brazil, respectively.

### Species-specific *Spiroplasma* prevalence patterns

**Figure 4:**
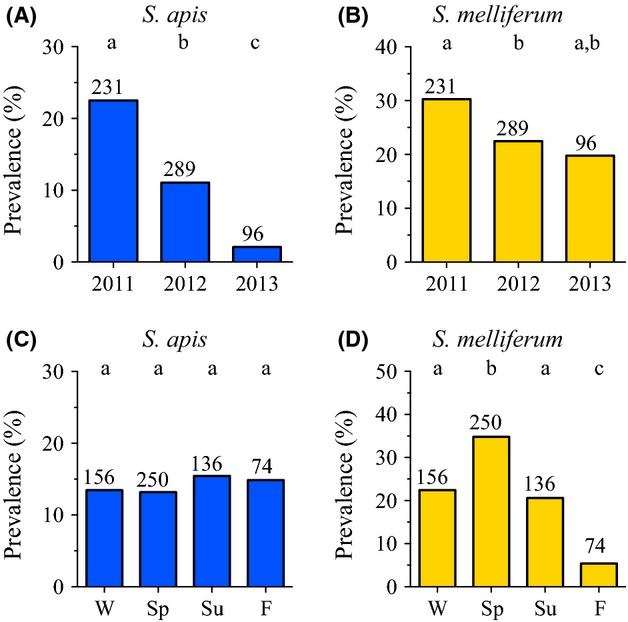
Summarized data collected from Maryland, U.S.A. *Apis mellifera* colonies between 2011 and 2013 show that each of the two honey bee *Spiroplasma* species have distinct annual and seasonal prevalence patterns. Species-specific oligonucleotide primers were used to identify and distinguish *S. apis* (A and C) from *S. melliferum* (B and D) using qPCR from pooled samples of adult worker honey bees. Significant differences in prevalence at *α* < 0.05 (indicated by columns with different letters) were determined with two-tailed Fisher's exact tests.

Between 2011 and 2013, *S. melliferum* (25.0%) was more prevalent overall than *S. apis* (14.0%) in the Unites States. The proportion of colonies infected by *S. apis* was highly variable year to year (*P* < 0.0001) but not seasonally (*P* = 0.9294; Table 1). In stark contrast, *S. melliferum* populations were more stable year to year (*P* = 0.0544) but showed highly significant seasonal variation (*P* < 0.0001). Contingency analyses showed significant decline in *S. apis* prevalence within U.S.A. colonies each year sampled (Table 2 and Fig. 4A), dropping by half between 2011 and 2012 (*P* = 0.0005) and by 81% between 2012 and 2013 (*P* = 0.0060). In contrast, *S. melliferum* dropped just significantly (*P* = 0.0449) by 26% between 2011 and 2012 and had a nonsignificant 12% drop (*P* = 0.6693) between 2012 and 2013 (Table 2 and Fig. 4B).

The significant increase in total *Spiroplasma* spp. (*S. apis* and/or *S. melliferum*) observed during spring in the U.S.A. (Fig. 3B and Table 1) was due solely to *S. melliferum* (Fig. 4D), which had significantly higher prevalence during spring (34.8%, mean = 42.2 ± 16.7% SEM) than any other season (winter *P* = 0.0103, summer *P* = 0.0036, fall *P* < 0.0001; Table 3). Prevalence of *S. melliferum* during fall (5.4%, mean = 5.1 ± 5.1% SEM) was significantly lower than any other season (winter *P* = 0.0011, spring *P* = <0.0001, summer *P* = 0.0042) while winter (22.4%) and summer (20.6%) did not differ significantly from one another. By comparison, *S. apis* did not contribute significantly to seasonal variation in prevalence (*P* = 0.5427 to *P* = 1.000, Fig. 4C and Table 3) and had lowest prevalence during spring (13.2%) compared to any other season (ranging from 13.5% to 15.4%).

**Figure 5:**
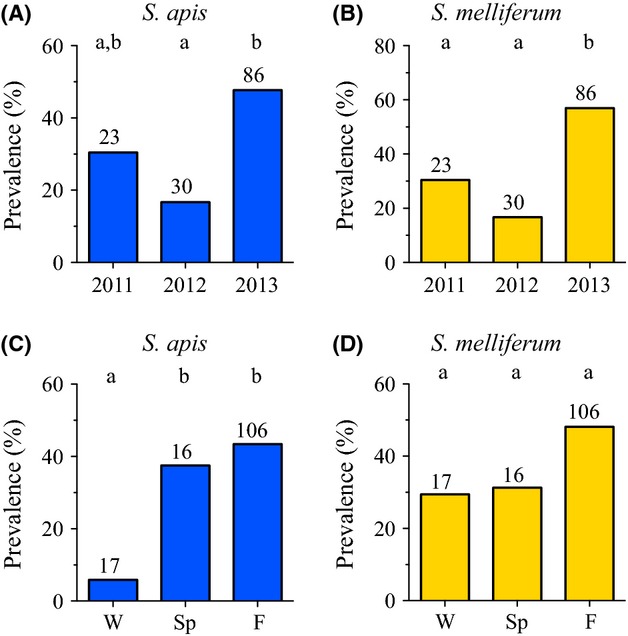
Species-specific qPCR *Spiroplasma* spp. data from *Apis mellifera* honey bee colonies sampled from 11 states in Brazil between 2011 and 2013 summarized by year and season. Both *S. apis* (A) and *S. melliferum* (B) showed similar yearly prevalence trends, where 2013 levels were significantly higher overall for each. Seasonal trends were different for each species. Fall and spring prevalence levels of *S. apis* (C) were significantly higher than winter levels. No significant seasonal variation in the prevalence of *S. melliferum* (D) occurred. Significance determined at *α* < 0.05 (indicated by columns with different letters) using two-tailed Fisher's exact tests.

Similar to overall U.S.A. data, *S. melliferum* predominated with respect to *S. apis* in Brazil at 43.8% versus 38.1% prevalence, respectively. Manifestly different, however, were the annual and seasonal species dynamics, where year to year prevalence (Table 1) was significantly variable for both *S. apis* (*P* = 0.0076) and *S. melliferum* (*P* = 0.0002). Comparisons of 2013 prevalence (Table 2 and Fig. 5A and B) showed that *S. apis* and *S. melliferum* were both more prevalent than 2012 samples (+31.0%, *P* = 0.0026; +40.3%, *P* = 0.0001) but only *S. melliferum* (+26.5%, *P* = 0.0338) was more prevalent than 2011 samples (Table 2). Identical trends in year to year significance were found in analyses delimited to SC and SP only colonies (Tables 1, 2).

Overall seasonal prevalence of *S. melliferum* was statistically stable (*P* = 0.1966) in Brazil colonies, while *S. apis* prevalence was significantly different (*P* = 0.0126; Table 1). The very low *S. apis* winter prevalence (5.9%) was significant compared to fall (43.4%, *P* = 0.0025) and spring (37.5%, *P* = 0.0391) levels (Table 3 and Fig. 5C). Analyses of *S. melliferum* (Fig. 5D) showed the noticeably higher fall prevalence (48.1%) was not significant compared to other seasons. In colonies from SC and SP only, however, *S. melliferum* prevalence in fall (51.1%) was significantly higher than winter (0%) (*P* = 0.0008; Table 3 and Table S3). Analyses of SC and SP only colonies corroborated the significantly low prevalence of *S. apis* in winter compared to spring (*P* = 0.0351) and fall (*P* = 0.0022) found using colony samples from all of Brazil.

### Spiroplasma co-infections versus single-species infections in honey bee colonies

**Figure 6:**
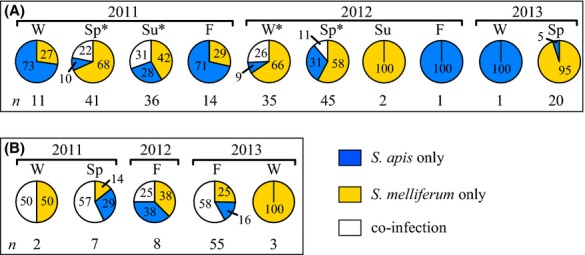
Summary of species composition from spiroplasma-infected *Apis mellifera* colonies from Maryland, U.S.A. (A) and 11 states in Brazil (B) sampled during 2011 through 2013. *Spiroplasma apis* only (blue), *S. melliferum* only (gold) or co-infection with both species (white). The percent of each infection type and the number of colonies infected with spiroplasma (*n*) are given by season for each year: winter (W), spring (Sp), summer (Su), fall (F). *Seasons with co-infections where mean prevalence of *Spiroplasma* spp. was significantly higher than mean prevalence from seasons when only single-species infections occurred (nonparametric two-tailed Mann–Whitney *U* test). Seasons in Brazil when no spiroplasma were detected are not shown.

Of all U.S.A. colony samples infected by *Spiroplasma* spp. (*n* = 206), 58.3% were infected only with *S. melliferum*, 25.2% only with *S. apis*, and 16.5% had co-infections of both species. Co-infection frequencies in the U.S.A. were relatively consistent from year to year (*P* = 0.0875) but significantly varied seasonally (*P* = 0.0015; Table 1) and occurred in seasons when mean *Spiroplasma* spp. prevalence was significantly higher (51.5 ± 22.1% SEM) compared to seasons when no co-infections occurred (16.0 ± 5.9% SEM; nonparametric two-tailed Mann–Whitney *U* test, *P* = 0.0190, indicated by asterisks in Fig. 6A). To test whether each species infected colonies in a mutually exclusive manner, two-tailed exact binomial probabilities were calculated from overall observed prevalence for each species (*S. apis* = 14.0%, *S. melliferum* = 25.0%). The observed frequency of co-infections (16.5%) was significantly higher than expected in the U.S.A. (*P* = 0.01397).

In comparison to the U.S.A., colonies in Brazil with *Spiroplasma* spp. (*n* = 75) had a significantly higher rate of co-infections (52.0%, *P* < 0.0001), significantly fewer *S. melliferum* only infections (29.3%, *P* < 0.0001), and statistically equal *S. apis* only infections (18.7%, *P* = 0.2702). Annual and seasonal co-infection prevalence patterns in Brazil did not reject the null hypothesis for equality across these factors (Table 1). As in the U.S.A., two-tailed exact binomial probabilities calculated from overall observed prevalence for each species in Brazil (*S. apis* = 38.1%, *S. melliferum* = 43.9%) revealed that co-infections (52.0%) occurred significantly more often than expected (*P* = 0.0011) in a statistically consistent manner over time (Fig. 6B). Delimited analyses from SC and SP only corroborated these conclusions (Table 1).

## Discussion

This study provides the first multiyear, population-level survey for both species of *Spiroplasma* known to infect adult European honey bees using a newly developed, high throughput qPCR diagnostic for each species. We also provide the first clear description that both *S. apis* and *S. melliferum* occur in Africanized honey bee hosts in Brazil. Previously, melanized hypopharyngeal glands of a honey bee presumably from Brazil (São Paulo) were found to contain unknown microbes with mollicute-like ultrastructure (Costa-Leonard and Silva de Moraes 1985), suggestive of the possible presence of *Spiroplasma* spp. in Brazil as early as 1985. Overall, our data show that spiroplasma populations fluctuate dramatically from year to year and season to season. Despite periods of low prevalence, honey bee colonies are likely reservoirs for both *S. apis* and *S. melliferum* throughout the year, answering a previously unknown question (Clark and Whitcomb 1984) relevant to the life history and population ecology of these species. We determined overall *Spiroplasma* spp. prevalence of 33% and 54% in the U.S.A. and Brazil, respectively, which supports that these are facultative (secondary) symbionts maintained dynamically by colonies and supports their exclusion from the obligate microbiota of honey bees (Gilliam 1997; Martinson et al. 2011). In light of recent global honey bee declines, these data reveal two facultative bacteria of interest to apiculture given their dynamic and sometimes prevalent occurrence in both North and South American honey bee colonies (shown here) and given their association with disease (shown previously).

### Seasonal variation of spiroplasmas in honey bee colonies

We have shown that honey bee colonies in both the U.S.A. and Brazil show seasonally variable rates of both *S. apis* and *S. melliferum*, although different seasons of peak prevalence appear to occur in the two countries. Both *Spiroplasma* species were detected in all four seasons sampled in the U.S.A. and all three seasons sampled in Brazil, and thus are not microbes limited to springtime or a single season as previously believed. In U.S.A. colonies, *S. melliferum* proved to be highly seasonal and significantly drove the overall observed *Spiroplasma* spp. seasonality. Our U.S.A. data corroborate previous work that directly links the epizootic appearance of *S. melliferum* to concurrent peak natural flower forage and colony population density (Winston 1992).

Spiroplasmas, including isolates believed to be identical or closely related to *S. apis* and *S. melliferum*, have been detected on various species of spring-flowering plants in the U.S.A. (Clark 1977; Raju et al. 1981). Plant species believed particularly important as likely transmission sites include members of the Magnoliaceae (Davis 1978; Raju et al. 1981) and Asteraceae (Mouches et al. 1982), both of which feature prominently as key forage sources for the Maryland colonies in our study including tulip poplar (Magnoliaceae; *Liriodendron tulipifera* L.) and a variety of Asteraceae (*Aster* spp. L. and *Solidago* spp. L.). Given the close association of spiroplasmas with flowers, conditions that affect annual flowering cycles may also affect spiroplasma levels in honey bee colonies. The 2012 *S. melliferum* peak prevalence period was unexpectedly early in Maryland, occurring during the late winter of 2012 (43.2%) and continuing into spring, but at lower frequency (23.5%). Ambient temperatures during this late winter period were well above average from January through March, with highs +4.6°F, +5.3°F, and +9.7°F above normal and lows +4.0°F, +4.8°F, and +8.6°F above normal (Fig. S5A). Further, concurrent lower than average precipitation (Fig. S5B) that led to an ongoing drought until October 2012 added to the atypical climatic conditions, and may have contributed to the surprisingly early *S. melliferum* peak by starting the spring flowering cycle earlier than normal. Honey bee flight activity was observed on unseasonably warm days beginning in late January of 2012 (R. S. Schwarz, pers. obs.) and a record of colony weight to track local nectar flows at one of the sampled apiaries in Maryland supports an earlier than normal spring flowering period in 2012 (Fig. S6). Similarly, the frequency of *S. apis* in Maryland also peaked in late winter of 2012 (16.2%), although this was only slightly higher than the subsequent spring period (14.4%). The degree to which *S. apis* utilizes flowers as a transmission site is less clear from previous research (Mouches et al. 1984) than is the case for *S. melliferum*. Our *S. apis* data show that minimal seasonal variation occurs for this species in the U.S.A. and supports a comparatively limited reliance upon seasonal flowers for their transmission or maintenance in honey bee colonies. Alternatively, it is also possible that the time between sample collections in spring of 2012 (30 March and 31 May) may have simply missed an ephemeral spring prevalence peak of these bacteria during the 2 months interim, which is plausible given the dynamic patterns we have shown here, particularly for *S. melliferum*. Nonetheless, the comparatively high wintertime prevalence levels of *S. apis* and *S. melliferum* in 2012 show that climate may be a factor that influences the appearance of these microbes in honey bee colonies.

Colony samples in Brazil encompassed broad geographies, which we accounted for in tandem analyses using a large subset of data from SC and SP states (*n* = 116), which are localized in latitude astride the Tropic of Capricorn in Brazil and differ in ∼5° of longitude, thus geographically delimited compared to additional samples from throughout Brazil (*n* = 23) collected from states that approached the equatorial border and spanned roughly 20° of longitude (Fig. S1). In contrast to the U.S.A., the observed significant seasonal fluctuations in *Spiroplasma* spp. across Brazil and in SC and SP only were significantly driven by *S. apis* and less so by *S. melliferum*. Although *S. melliferum* did not contribute quite significantly to seasonality in Brazil overall, it shared significant contribution to seasonal variation with *S. apis* when data analyses were restricted to SC and SP only. This highlights the important consideration that significant seasonal changes in *Spiroplasma* spp. prevalence can be associated with local conditions. Seasonal availability of flower resources are likely to be much more similar within narrowed geographic regions. The significant seasonality in *S. melliferum* (winter vs. fall) and *Spiroplasma* spp. overall (winter vs. spring) that uniquely became apparent in our analyses of samples from SC and SP only compared to samples from all 11 states of Brazil support the point that local conditions (i.e., plant communities and flowering cycles) may regulate seasonal transmission and corresponding prevalence levels of *Spiroplasma* species in local honey bee colonies. The main vegetation types of collection sites in SC and SP were Atlantic forest, Araucaria forest, Ombrophilous forest, steppe and coastal vegetation, with mean annual temperatures ranging from 14 to 22°C.

### Annual variation in spiroplasma populations within honey bee colonies

Colonies in both the U.S.A. and Brazil had strong year to year variation of *S. apis* and *S. melliferum* prevalence during 2011 to 2013. The causes behind such fluctuations are not known, but climatic events may play a role as discussed above. We have shown that in the U.S.A., *S. apis* most strongly drives year to year variation of spiroplasma populations but does not show significant seasonal changes. Conversely, *S. melliferum* shows strong seasonality and comparatively less variation across years (via chi-square analyses), although individual year comparisons (Fisher's exact tests) revealed that annual variation in *S. melliferum* populations may occur, albeit to a less significant extent. In Brazil, both *Spiroplasma* species contributed significantly to annual variation while *S. apis* alone was responsible for significant seasonal variation, a clear contrast to the patterns observed in the U.S.A. Taken together, these data support that *S. apis* and *S. melliferum* have distinct life history dynamics from one another within honey bee colonies living: (1) in a contiguous, limited range of temperate geography (Maryland) and (2) in distinct geographic locations (U.S.A. vs. Brazil). These findings support that *S. apis* and *S. melliferum* may be regionally significant microbes within honey bee colonies, each with their own unique patterns of prevalence that may depend on temporal, geographic, and climatic variables.

### The role of spiroplasma infections in honey bee health

Although the nature of their relationship (mutualism to pathogenic) as facultative symbionts in honey bees cannot yet be clearly described, previous work suggests sporadic pathogenicity is caused by *S. apis*, with some years severe and others minor (Mouches et al. 1984). Experimentally, both *S. apis* (Mouches et al. 1982) and *S. melliferum* (Clark 1982) cause systemic infections and increased mortality in honey bees. While the direct impact of spiroplasmas on honey bee immune competence is unknown, studies with *Drosophila* (Eleftherianos et al. 2013) suggest *Spiroplasma* are not directly immunogenic but can dampen host immune responses, increasing host susceptibility to additional microbes. Given the wide variety of *Spiroplasma*-host associations (Fig. 1), however, this conclusion should not be extrapolated to other species without empirical study.

The multiyear variation in *S. apis* and *S. melliferum* we have shown here provides a congruous explanation for the disparate disease cycles observed in the past and support that these species may be unrecognized pathogens involved in modern disease cycles of honey bees that must be considered under the novel context of at least three newly emergent parasites not believed to be present at the time of original honey bee spiroplasma investigation: *Nosema ceranae* fungus, *Varroa destructor* mite, and Varroa Destructor Virus-1. Health impacts to a honey bee colony infected with both *Spiroplasma* species (as shown here) may be compounded if additional parasites concurrently (e.g., Cornman et al. 2012) or subsequently (Hedtke et al. 2011) infect the colony, as evidenced by altered immune responses in honey bees during mixed-species infections (Schwarz and Evans 2013). Factors involved in honey bee spiroplasma virulence are untested, but candidate cell adhesion and invasion proteins (Yu et al. 2000; Killiny et al. 2005; Alexeev et al. 2012; Béven et al. 2012; Duret et al. 2014) identified from other species of *Spiroplasma* may similarly be used by *S. apis* and *S. melliferum* to infect honey bees.

### Approaches to detecting and assessing spiroplasma populations

Unlike the commonly studied bacterial pathogens in honey bees that cause the readily recognized and diagnosed pathologies of foulbrood diseases (*Paenibacillus larvae* and *Melissococcus plutonius*), spiroplasma-infected bees are impossible to identify by outward pathology. Honey bee disease diagnostic laboratories that we are aware of do not routinely, if ever, screen for *S. apis* or *S. melliferum* via hemolymph cultivation (as was done historically) nor using a multiplex PCR technique made recently available (Meeus et al. 2012). In addition, the need for specialized dark-field microscopy to visualize the bacteria, and the fact that infected adults may die unnoticed away from the hive help explain the gap in knowledge regarding spiroplasmas with today's bee keepers and researchers. Our approach to implement a highly sensitive qPCR assay for the detection of *S. apis* and *S. melliferum* is based on the confirmed utility and common use of qPCR in large-scale honey bee health surveys (Evans et al. 2013). The detection methods we describe are readily transferrable to such surveys where RNA or DNA templates are extracted from samples, and may be used quantitatively or qualitatively.

Prior honey bee microbe deep sequencing and metagenomic surveys (Cox-Foster et al. 2007; van Engelsdorp et al. 2009; Cornman et al. 2012) may have missed detecting *S. melliferum* and *S. apis* via read mapping due to the lack of assembled genomes at the time, and may have been unlikely to assemble *Spiroplasma* spp. reads de novo given their ephemeral and sometimes very low to absent levels in colonies. Assembled *S. melliferum* (Alexeev et al. 2012; Lo et al. 2013) and *S. apis* (Ku et al. 2014) genomes now available will make the identification of these species by metagenomic surveys more likely.

### Other potential hosts to *S. apis* and *S. melliferum*

Our conclusion that honey bees act as long-term reservoirs for *S. apis* and *S. melliferum* does not rule out the possibility that additional reservoirs may exist and contribute to their life cycle. If alternate or incidental hosts (other pollinators visiting the transmission site) exist, pathogenicity may be affected (likely attenuated). Incidental infections of *S. melliferum* to other Hymenoptera include solitary bees (*Andrena* sp. and *Anthophora abrupta*), bumble bees (*Bombus impatiens, Bombus pennsylvanicus, Bombus pascuorum*), and particularly the Japanese hornfaced bee (*Osmia cornifrons*) (Clark and Whitcomb 1984; Clark et al. 1985; Meeus et al. 2012). It has even been isolated from more diverse hosts including robber fly (Asilidae) and butterfly (Papilionoidea) (Clark et al. 1985). Aside from honey bee, *S. apis* has so far only been detected in a European bumble bee (*B. pratorum*) (Meeus et al. 2012) to our knowledge, although survey efforts for *S. apis* in other species have been minimal. Whether these alternate host infections are due to incidental spillover or indicate additional reservoirs for the bacteria remains unknown.

## Conclusions

This study helps to explain how *Spiroplasma* spp. populations are maintained by honey bee colonies from year to year by showing both *S. apis* and *S. melliferum* occur in all seasons from temperate climates in the U.S.A. and all seasons tested (winter, spring, fall) from tropical/subtropical climates in Brazil. Both species contribute significantly to annual variation in *Spiroplasma* spp. prevalence while seasonal variation is driven by different species compositions in the U.S.A. compared to Brazil. These results clarify two important variables, time and geography, that contribute significantly to the life history dynamics of *Spiroplasma* spp. populations in honey bee colonies. Our finding that *S. apis* and *S. melliferum* establish co-infections more frequently than expected could be due to several factors, including shared transmission routes via key flower species and reduced host immune competence that makes bees more susceptible to one species when already infected by the other. The higher rate of co-infections in Africanized honey bees from Brazil may reflect the availability of more consistent flower resources in the habitats of Brazil that facilitate the transmission cycle of both species. Finally, our comparison of data from across the longitudinal gradient in Brazil to more delimited data from SC and SP highlight the important need to consider the role of geography in understanding regionally specific pathogen dynamics.

## Supporting Information

**Figure S1.** Location of 11 states throughout Brazil from which honey bee colonies were sampled for this study between 2011 and 2013. CE – Ceará, RN – Rio Grande do Norte, BA – Bahia, GO – Goiás, MS – Mato Grosso do Sul, MG – Minas Gerais, RJ – Rio de Janeiro, SP – São Paulo, PR – Paraná, SC – Santa Catarina, and RS – Rio Grande do Sul. Locations of colonies qPCR positive for spiroplasmas are indicated on the map as follows: • *S. apis. ♦ S. melliferum*. ▲ *S. apis and S. melliferum* co-infection.

**Figure S2.** Alignment of the SSU rRNA and ITS-1 region from members of the Apis and Citri spiroplasma clades used to design the *S. apis* species-specific primer “*S. apis* ITS.” Forward and reverse primer sites are boxed in red. Three strains of *S. melliferum* and the type strain of *S. apis* (B31) are in bold font and GenBank accession numbers are given for all sequences. Genome sequences between the primer regions of the alignment were removed for space and are indicated by “//”. Regions of the alignment with 100% nucleotide identity are shaded in gray and gaps introduced for alignment purposes by a hash mark. “*S. apis* ITS forward”: 5′-AATGCCAGAAGCACGTATCC-3′ and “*S. apis* ITS reverse”: 5′-GAACGAGATATACTCATAAGCTGTTACAC-3′.

**Figure S3.** Spiroplasma amplicons produced following qPCR with “*S. apis* ITS” (lanes 1, 2, and 3) and “Ms-160” (lanes 4, 5, and 6) primer sets and analyzed by gel electrophoresis. A marker (M) shows reference nucleic acid fragment sizes.

**Figure S4.** Spiroplasma primer results from qPCR analysis. (A) Standard curves and genome copy number detection ranges for each primer used in this study based on known dilutions of recombinant plasmids containing the target. Linear equations from the dilution series for each primer were automatically calculated in multiple runs, where *b* = y intercept, *m* = slope, and *E* = primer efficiency, and ranged as follows: “*S. apis* ITS”: *m* = −3.395 to −3.621, *b* = 46.281–50.862, *R*^2^ = 0.974–0.997, *E* = 88.9–97.0%; “Ms-160”: *m* = −3.257 to −3.564, *b* = 44.648–48.364, *R*^2^ = 0.985–0.994, *E* = 90.8–102.8%. (B) Melt curves for amplicons generated from “Ms-160” (78°C) and “*S. apis* ITS” (81.5°C) show amplicon intensity given as the negative derivative of the relative fluorescence units (RFU) per temperature (*y*-axis) and dissociation temperature of the double-stranded amplicons (*x*-axis). *Note*: Please refer to “Material and Methods” for details on qPCR reagents and equipment used. As described in “Material and Methods,” amplicon melt temperatures may vary slightly for researchers using different chemistry and/or equipment and will require empirical confirmation.

**Figure S5.** Weather data from Beltsville, MD between 2011 and 2013 showing monthly mean maximum and minimum ambient temperatures (A) and monthly mean precipitation (B). The late winter period, during which 2012 spiroplasmas showed an unexpectedly early peak, are highlighted and show 2012 above normal temperatures and below normal precipitation. Weather data obtained from the National Climate Data Center at http://www.ncdc.noaa.gov/oa/ncdc.html. Station location used: GHCND:USC00180700.

**Figure S6.** Nectar flow data at Beltsville, MD from 2010 (A), 2011 (B), and 2012 (C) using scale hive data (Fairbanks Model 1124, 0.5 lb divisions) on an *Apis mellifera ligustica* nonmigratory colony (two deeps plus supers). By the beginning of spring (day 80) in 2012, nectar flow was already strong enough to have allowed the colony to increase their weight by approximately 2 pounds (red line: normalized cumulative weight). Typical colony weight loss throughout late winter and into early spring is exemplified by 2010 (A) and 2011 (B) data from this same colony. Daily (blue line) and weekly (green line) change in weight are also shown for reference. *Note*: 2013 data were not available for this site due to death of the colony. Data from and available at http://honeybeenet.gsfc.nasa.gov.

**Table S1**. *Spiroplasma apis* and *Spiroplasma melliferum* amplicon sequences obtained with the qPCR primers used in this study.

**Table S2.** *Apis mellifera* honey bee colonies surveyed for *Spiroplasma* species using qPCR analysis in Brazil from all seasons and separated by state. BA – Bahia, CE – Ceará, GO – Goiás, MG – Minas Gerais, MS – Mato Grosso do Sul, PR – Paraná, RJ – Rio de Janeiro, RN – Rio Grande do Norte, RS – Rio Grande do Sul, SC – Santa Catarina, and SP – São Paulo.

**Table S3.** Annual data by season of spiroplasma (*S. apis* and *S. melliferum*) prevalence in U.S.A. and Brazil *Apis mellifera* honey bee colonies between 2011 and 2013 based on species-specific qPCR results.
