# Supplementary material for: Honey bee colonies act as reservoirs for two Spiroplasma facultative symbionts and incur complex, multiyear infection dynamics
Source: Microbiologyopen. 2014 Apr 28;3(3):341–55. doi: 10.1002/mbo3.172 (PMC4082708; doi:10.1002/mbo3.172)
Supplement: Supplementary file 2 — Figure S2. Alignment of the SSU rRNA and ITS-1 region from members of the Apis and Citri spiroplasma clades used to design the S. apis species-specific primer “S. apis ITS.” Forward and reverse primer sites are boxed in red. Three strains of S. melliferum and the type strain of S. apis (B31) are in bold font and GenBank accession numbers are given for all sequences. Genome sequences between the primer regions of the alignment were removed for space and are indicated by “//”. Regions of the alignment with 100% nucleotide identity are shaded in gray and gaps introduced for alignment purposes by a hash mark. “S. apis ITS forward”: 5′-AATGCCAGAAGCACGTATCC-3′ and “S. apis ITS reverse”: 5′-GAACGAGATATACTCATAAGCTGTTACAC-3′. [file mbo30003-0341-sd2.docx]

10 20 30 40 50 60 //

Apis

Citri

....|....|....|....|....|....|....|....|....|....|....|....|

S.syrphidicola_EA-1_DQ439673 CGCCCGTCACACCATGAGAGTTGATAATACCAGAAGTCGGTATCCTAACCGTAAGGAGGG

S.insolitum_M55_DQ004918 CGCCCGTCACACCATGAGAGTTGATAATACCAGAAGTCGGTATTCTAACCGTAAGGAGGA

S.poulsonii_DW-1_AY973574 CGCCCGTCACACCATGAGAGTTGATAATACCAGAAGTCGGTATTCTAACCGCAAGGAGGA

**S.melliferum_F316_DQ004904** CGCCCGTCACACCATGAGAGTTGTTAATACCAGAAGTCGGTATTCTAACCGCAAGGAGGA

**S.melliferum KC3_DQ004906** CGCCCGTCACACCATGAGAGTTGATAATACCAGAAGTCGGTATTCTAACCGCAAGGAGGA

**S.melliferum BC-3_DQ004905** CGCCCGTCACACCATGAGAGCTGATAATACCAGAAGTCGGTATTCTAACCGCAAGGAGGA

**S.apis_B31_AY736030** CGCCCGTCACACCATGAGAGTTGGTAATGCCAGAAGCACGTATCCTAACCGTAAGGAGGG

S.montanense_HYOS-1_DQ004925 CGCCCGCCACACCATGAGAGTTGGTAATGCCAGAAGCACGTATCCTAACCGTAAGGGGGG

S.leptinotarsae_LD-1_AY772216 CGCCCGTCACACCATGAGAGTTGGTAATACCAGAAGTATGTATTCTAACCGTAAGGAGGA

S.sabaudiense_AR1343_DQ004907 CGCCCGTCACACCATGAGAGTTGGTAATACCAGAAGCAGGTGTCCTAACCGTAAGGAGGG

S.alleghenense_PLHS-1_DQ004922 CGCCCGTCACACCATGAGAGTTGGTAATACCAGAAGCAGGTGTCCTAACCGTAAGGAGGG

S.lampyridicola_PUP-1_AY770625 CGCCCGTCAAACCATGAGAGTTGATAATGCCAGAAGTACGTATCTTAACCGTAAGGAGGG

S.taiwanense_CT-1_DQ439667 CGCCCGTCACACCATGAGAGTTGGTAATACCAGAAGCACGTATCTTAACCGTAAGGAGGG

S.cantharicola_CC-1_DQ861914 CGCCCGTCACACCATGAGAGTTGGTAATACCAGAAGCACGTATCCTAACCGTAAGGAGGG

S.diminutum_CUAS-1_DQ004920 CGCCCGTCACACCATGAGAGTTGGTAATACCAGAAGCACGTATCTTAACCGTAAGGAGGG

S.monobiae_MQ-1_DQ004914 CGCCCGTCACACCATGAGAGTTGGTAATACCAGAAGCACGTATCTTAACCGTAAGGAGGG

S.floricola_23-6_AY729931 CGCCCGTCACACCATGAGAGTTGGTAATACCAGAAGCACGTATCTTAACCGTAAGGAGGG

S.diabroticae_DU-1_GU908490 CGCCCGTCACACCATGAGAGTTGGTAATACCAGAAGCACGTATCTTAACCGTAAGGAGGG

S.corruscae_EC-1_DQ004943 CGCCCGTCACACCATGAGAGTTGGTAATACCAGAAGCACGTATCTTAACCGCAAGGAGAG

S.turonicum_Tab-4c_DQ439669 CGCCCGTCACACCATGAGAGTTGGTAATACCAGAAGCACGTATCTTAACCGTAAGGAGAG

S.clarkii_CN-5_AY772218 CGCCCGTCACACCATGAGAGTTGGTAATACCAGAAGCACGTATCTTAACCATTAGGAGAG

S.velocicrescens_MQ-4_DQ439666 CGCCCGTCACACCATGAGAGTTGGTAATACCAGAAGCACGTATCTTAACCGTAAGGAGAG

S.culicicola_AEF-1_AY780799 CGCCCGTCACACCATGAGAGTTGGTAATACCAGAAGCATGTATCTTAACCGCAAGGAGAG

S.helicoides_TABS-2_DQ004903 CGCCCGTCACACCATGAGAGTTGGTAATACCAGAAGCACGTATCTTAACCGTAAGGAGAG

S.tabanidicola_TAUS-1_DQ004931 CGCCCGTCACACCATGAGAGTTGGTAATACCAGAAGCACGTATCTTAACCGTAAGGAGAG

S.lineolae_TALS-2_DQ860100 CGCCCGTCACACCATGAGAGTTGGTAATACCAGAAGCACGTATCTTAACCGTAAGGAGAG

S.gladiatoris_TG-1_DQ004899 CGCCCGTCACACCATGAGAGTTGGTAATGCCAGAAGCACGTATCTTAACCGTAAGGAGAG

Apis

Citri

// 190 200 210 220 230 240

....|....|....|....|....|....|....|....|....|....|....|....|

S.syrphidicola_EA-1_DQ439673 --------------------TAAA---------TACGG-CTATAATGAA-GTTATGTTTA

S.insolitum_M55_DQ004918 --------------------TAATTTAATTAGTTTTAA-TGACC------GTTATGTTTA

S.poulsonii_DW-1_AY973574 --------------------TAATCTAATTAGTTTTAA-TGACC------GTTATGTTTA

**S.melliferum_F316_DQ004904** --------------------TA-ATTAACTAGTTTTAA-TGACC------GTTATGTTTA

**S.melliferum KC3_DQ004906** --------------------TA-ATTAACTAGTTTTAA-TGACC------GTTATGTTTA

**S.melliferum BC-3_DQ004905** --------------------TA-ATTAACTAGTTTTAA-TGACC------GTTATGTTTA

**S.apis_B31_AY736030** AAGCTG--AGCGAATCGG--TG---TAACAGCTTATGA-GTATATCTC--GTTCTATCTA

S.montanense_HYOS-1_DQ004925 AAGCTG--AGCGAATCGG--TG---TAACAGCTTATGG-GTATATCTC--GTTCTATCTA

S.leptinotarsae_LD-1_AY772216 CAGTT---AATGAAATTATGTACATAAGCATTGTTCGG------------GTTCTATAAA

S.sabaudiense_AR1343_DQ004907 AAGCTGAAAGTGACTAAG--TGTATTAACAGCTT-CGA-CTATCTTTTATGTTCTATTTA

S.alleghenense_PLHS-1_DQ004922 AAGCTGAAAGTGACTAAG--TGTATTAACAGCTT-CGA-CTATCCTTTATGTTCTATTTA

S.lampyridicola_PUP-1_AY770625 AAGCTG------AAGTGA--TA-ATTAACAGCTTTCAA-TTCTACTTGA-GTTCTATCTA

S.taiwanense_CT-1_DQ439667 AAGCTG--TGTGAAAT-A--TA-A--AACAGCTTTTGG-TTATATCTAA-GTTCTATCTA

S.cantharicola_CC-1_DQ861914 AAGCTG--AGTGAAACGA--TA-A--AACAGCTTTTGA-TTATATCT-C-GTTCTATCTA

S.diminutum_CUAS-1_DQ004920 AAGCTG--AGTGAAACGA--TA-A--AACAGCTTATGA-TTATATCT-C-GTTCTATCTA

S.monobiae_MQ-1_DQ004914 AAGCTG--AGTGAAACGA--TA-A--AACAGCTTATGA-TTATATCT-C-GTTCTATCTA

S.floricola_23-6_AY729931 AAGCTG--AGTGAAACGA--TA-A--AACAGCTTATGA-TTATATCT-C-GTTCTATCTA

S.diabroticae_DU-1_GU908490 AAGCTG--AGTGAAACGA--TA-A--AACAGCTTATGA-TTATATCT-C-GTTCTATCTA

S.corruscae_EC-1_DQ004943 AAGCTG--AGTGAAAA----TG-ATTAACAGCTTATGGCTTATATCTTG-GTTCTATCTA

S.turonicum_Tab-4c_DQ439669 AAGCTG--AGTGAA-CGA--TA-A--AACAGCTTTTGGTTTATATCTTA-GTTCTATCTA

S.clarkii_CN-5_AY772218 AAGCTG--AGCGAATCGA--TA-A--AACAGCTTTTGA-TTATATTCTG-GTTCTATCTA

S.velocicrescens_MQ-4_DQ439666 AAGCTG--AGCGAATCGA--TA-A--AACAGCTTTTGA-TTATATCTC--GTTCTATCTA

S.culicicola_AEF-1_AY780799 AAGCTG--AGCGAATCGA--TA-A--AACAGCTTTTGA-TTATATTTG--GTTCTATCTA

S.helicoides_TABS-2_DQ004903 AAGCTG--AGCGAATCGA--TA-A--AACAGCTTATGA-CTAAATCTG--GTTCTATCTA

S.tabanidicola_TAUS-1_DQ004931 AAGCTG--AGCGAATCGA--TA-A--AACAGCTTTTGA-TTATATTCTG-GTTCTATCTA

S.lineolae_TALS-2_DQ860100 AAGCTG--AGCGAATCGA--TA-A--AACAGCTTTTGA-TTATATTCTG-GTTCTATCTA

S.gladiatoris_TG-1_DQ004899 AAGCTG--AGCGAATCGA--TA-A--AACAGCTTTTGG-TTATAATCTG-GTTCTATCTA

**Figure S2** Alignment of the SSU rRNA and ITS-1 region from members of the Apis and Citri spiroplasma clades used to design the *S. apis* species-specific primer “S. apis ITS”. Forward and reverse primer sites are boxed in red. Three strains of *S. melliferum* and the type strain of *S. apis* (B31) are in bold font and GenBank accession numbers are given for all sequences. Genome sequences between the primer regions of the alignment were removed for space and are indicated by “//”. Regions of the alignment with 100% nucleotide identity are shaded in gray and gaps introduced for alignment purposes by a hash mark. “S. apis ITS forward”: 5’- AATGCCAGAAGCACGTATCC -3’ and “S. apis ITS reverse”: 5’- GAACGAGATATACTCATAAGCTGTTACAC -3’.
