# Supplementary material for: Honey bee colonies act as reservoirs for two Spiroplasma facultative symbionts and incur complex, multiyear infection dynamics
Source: Microbiologyopen. 2014 Apr 28;3(3):341–55. doi: 10.1002/mbo3.172 (PMC4082708; doi:10.1002/mbo3.172)
Supplement: Supplementary file 7 — Table S1. Spiroplasma apis and Spiroplasma melliferum amplicon sequences obtained with the qPCR primers used in this study. [file mbo30003-0341-sd7.docx]

**Table S1**: *Spiroplasma apis* and *Spiroplasma melliferum* amplicon sequences obtained with the qPCR primers used in this study.

| **Primer ID** | **Nucleotide sequence of amplicon** |
| --- | --- |
| S. apis ITS forward + reverse | AATGCCAGAAGCACGTATCCTAACCGTAAGGAGGGAGCGTACCAAGGTAGGATTAGCGATTAGGGTGAAGTCGTAACAAGGTATCCGTACGGGAACGTGCGGATGGATCACCTCCTTTCTATGGAGTATCATAGTTTAGAGAAAAAAAGCTGAGCGAATCGGTGTAACAGCTTATGAGTATATCTCGTTC |
| Ms-160 forward + reverse | TTGCAAAAGCTGTTTTAGATGCTATACAAAAGATAGCACCAAATGCAGGAGCAAGTGATTTTGAAATTACAAATAATGGTGCTGAGGGAGACTATGAAGCTGCAAAAGAAGTTGAAGTAACAGTTAAAGCAAAAAATGATTCAGCAAACATTTCTGGTCA |

Schwarz RS, Weinstein Teixeira E, Tauber JP, Birke JM, Martins M, Fonseca I, Evans JD. 2013. Honey bee colonies act as reservoirs for two *Spiroplasma* facultative symbionts and incur complex, multiyear infection dynamics.
